# Synthesis and crystal structure of a new polymorph of potassium europium(III) bis(sulfate) mono­hydrate, KEu(SO_4_)_2_·H_2_O

**DOI:** 10.1107/S2056989018000567

**Published:** 2018-01-26

**Authors:** Avijit Kumar Paul

**Affiliations:** a Department of Chemistry, National Institute of Technology Kurukshetra, Haryana 136119, India

**Keywords:** crystal structure, polymorphism, topological analysis

## Abstract

The mixed-metal sulfate, KEu(SO_4_)_2_·H_2_O, has been obtained as a new polymorph using hydro­thermal conditions. The crystal structure is isotypic with NaCe(SO_4_)_2_·H_2_O and shows a three-dimensional connectivity of the tetra­hedral sulfate units with Eu^III^ and K^I^ ions. Tricapped trigonal–prismatic EuO_9_ units and square-anti­prismatic KO_8_ units link the SO_4_ tetra­hedra, building the three-dimensional structure. Topological analysis reveals the existence of two nodes with 6- and 10-connected nets. The compound was previously reported [Kazmierczak & Höppe (2010 ▸). *J. Solid State Chem.* **183**, 2087–2094] in the monoclinic space group *P*2_1_/*c* with a similar structural connectivity and coordination environments to the present compound.

## Abstract 2

In comparison with the known polymorph of KEu(SO_4_)_2_·H_2_O (monoclinic, *P*2_1_/*c*), the new polymorph crystallizes in the trigonal crystal system (space group *P*3_1_21).

## Chemical context   

The design of new solids including rare earth metal ions is an emerging field because of their potential applications in catalysis, luminescence and optoelectronics (Ramya *et al.*, 2012 ▸; Höppe, 2009 ▸; Mahata *et al.*, 2008 ▸; Shehee *et al.*, 2003 ▸). In general, the discovery of new solids is a major thrust in the field of solid-state research because of their diverse topol­ogical architectures and properties. In particular for rare earth metal compounds, the connectivity within the crystal structure becomes novel and complex as the coordination numbers are higher than for transition metals. In this regard, crystal engin­eering becomes challenging with non-centrosymmetric solids as it can lead to many chiral-related applications such as enanti­oselective separation, heterogeneous chiral catalysis or non-linear optical (NLO) effects (Ramya *et al.*, 2012 ▸; Höppe, 2009 ▸; Mahata *et al.*, 2008 ▸; Shehee *et al.*, 2003 ▸; Halasyamani & Poeppelmeier, 1998 ▸; Sweeting & Rheingold, 1987 ▸). Obtaining new structures with various anions such as silicates, phosphates, phosphites, carboxyl­ates, sulfates, arsenates, selenates, selenites, germanates, borates or thio­sulfates is a long-standing research area (Sweeting *et al.*, 1992 ▸; Paul, 2016 ▸; Paul & Natarajan, 2010 ▸; Paul *et al.*, 2009 ▸, 2010 ▸; Natarajan & Mandal, 2008 ▸; Natarajan *et al.*, 2006 ▸; Feng *et al.*, 2005 ▸; Hathwar *et al.*, 2011 ▸; Held, 2014 ▸). A rare earth metal can be a better choice than a transition metal as it provides many variations arising from coordination preferences, ligand geometry and valence states. The presence of two metals in a crystal structure can introduce more structural variation along with specific properties. From earlier reports, it is obvious that the design of chiral frameworks mostly require chiral fragments or chiral ligands. The synthesis of sulfate compounds with a chiral framework is a challenging task that requires a particular strategy. Hence, the synthetic strategy was modified (piperazine was used, which is not in the product but supports the crystallization of the sulfate compound) and the resultant compound is a new polymorph of KEu(SO_4_)_2_·H_2_O that is isotypic with trigonal NaCe(SO_4_)_2_·H_2_O (Blackburn & Gerkin, 1995 ▸).

## Structural commentary   

**Figure 1:**
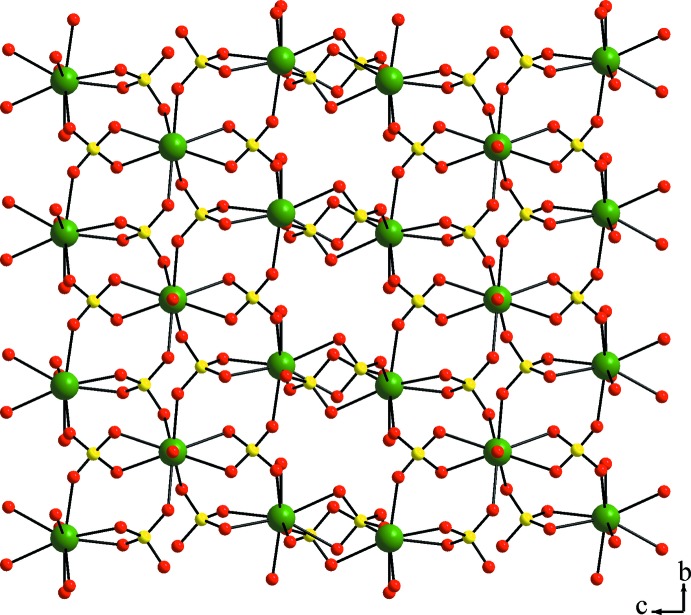
Three-dimensional framework observed by connectivity between the Eu^III^ ions and the SO_4_^2−^ units. Green, yellow and red spheres represent Eu, S and O sites, respectively.

**Figure 2:**
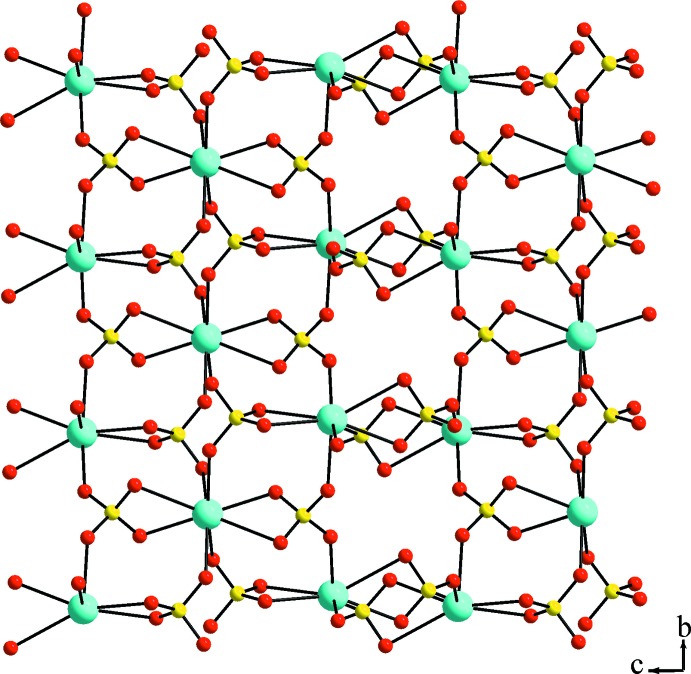
Three-dimensional framework observed by connectivity between the K^I^ ions and the SO_4_^2−^ units. Cyan, yellow and red spheres represent K, S and O sites, respectively.

**Figure 3:**
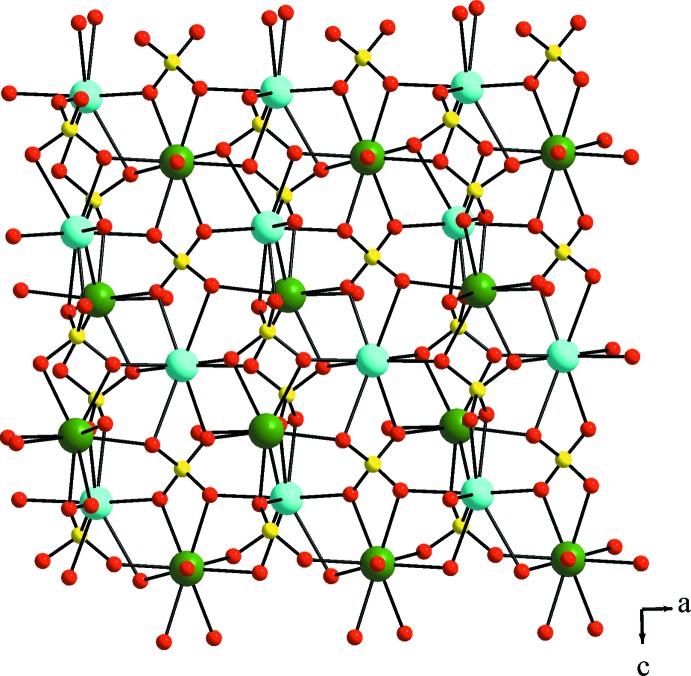
Overall three-dimensional connectivity between the Eu^III^ ions, the K^I^ ions and the SO_4_^2−^ units. Green, cyan, yellow and red spheres represent Eu, K, S and O sites, respectively

The asymmetric unit of trigonal KEu(SO_4_)_2_·H_2_O contains eight non-hydrogen atoms, of which one Eu, one K and one O site (defining the water mol­ecule) are located on a twofold rotation axis, and one complete sulfate unit. The Eu^III^ ion is coordinated by the O atoms of six sulfate tetra­hedra (two chelating, four in a monodentate way) and one water mol­ecule in a tricapped-trigonal–prismatic environment. The Eu—O bond lengths range from 2.425 (4) to 2.518 (4) Å with an average of 2.469 Å. The resulting three-dimensional Eu/SO_4_ framework is displayed in Fig. 1 ▸. The K^I^ ion is eight-coordin­ated by six sulfate units, again two chelating and four in a monodentate way, leading to a square-anti­prismatic KO_8_ coordination polyhedron with K—O distances ranging from 2.374 (5) to 2.830 (4) Å and an average of 2.556 Å. The K^I^ ions form a similar three-dimensional potassium sulfate framework (Fig. 2 ▸). The sulfate ion is an almost regular tetra­hedron with S—O distances ranging from 1.456 (4) to 1.484 (4) Å and O—S—O angles of 105.2 (2)–112.4 (3)°. The overall three-dimensional connectivity between the two metal cations and the sulfate anions is given in Fig. 3 ▸. The present framework structure crystallizes isotypically with NaCe(SO_4_)_2_·H_2_O (Blackburn & Gerkin, 1995 ▸). It should be noted that the reported structure of NaEu(SO_4_)_2_·H_2_O (Wu & Liu, 2006 ▸) shows the same space-group type, very similar lattice parameters, and unexpectedly also very similar Na—O distances in comparison with the K—O distances of the title compound. The previously reported KEu(SO_4_)_2_·H_2_O polymorph crystallizes in space group *P*2_1_/*c* (Kazmierczak & Höppe, 2010 ▸) and in comparison shows a similar connectivity and respective coordination polyhedra.

## Supra­molecular features   

**Figure 4:**
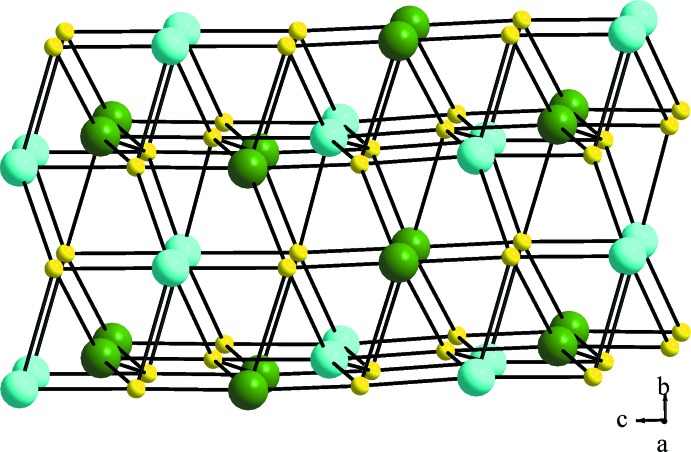
Three-dimensional node connectivity of the title compound. Green, cyan and yellow spheres represent the Eu and K sites and the SO_4_ unit, respectively.

As the hydrogen-atom positions could not be located during the present study, hydrogen-bonding inter­actions are not discussed here. An inter­esting structural feature arises due to the formation of three kinds of helices along the 3_1_ screw axes. A detailed structural analysis of the topology of the framework was performed using *TOPOS* (Blatov *et al.*, 2014 ▸). The EuO_9_, KO_8_ and SO_4_ polyhedra are considered as different nodes and represented in different colors (Fig. 4 ▸). Although the potassium and europium cations have different coordination environments, both have similar coordination behaviors, with terminal water only extra for europium. In topological terms, both form similar 10-connected nets with three-, four-, five- and six-membered rings, point symbol 3^12^.4^14^.5^12^.6^7^. The sulfate unit is associated with three-, four- and five-membered rings and forms a 6-connected net with point symbol 3^6^.4^6^.5^3^. The topological approach thus allows the present complex structure to be visualized in a different way by considering the node-connectivity.

## Synthesis and crystallization   

The title compound was synthesized under hydro­thermal conditions. All chemicals were purchased from Aldrich and used without further purification. Eu(COOCH_3_)_3_·*x*H_2_O (0.329 g, 1 mmol) was dissolved in 10 ml water. Then K_2_SO_4_ (0.348 g, 2 mmol) was added to the solution, which was stirred for 30 mins. Finally, piperazine (0.043 g, 0.5 mmol) was added to the reaction mixture and the pH was observed to be 8. The entire mixture was stirred for another 30 mins and poured into a 23 ml Teflon-lined autoclave. The autoclave was kept at 426 K for 5 d. The product was then filtered off and washed with water. The product contained some block-like single crystals accompanied with a light-yellow powder. The yield was approximately 75% based on Eu metal.

## Refinement   

Crystal data, data collection and structure refinement details are summarized in Table 1 ▸. The correctness of all atom types was checked by free refinement of the occupancy. Hydrogen atoms of the lattice water mol­ecule could not be located in difference-Fourier maps. If the hydrogen atoms were included in calculated positions and refined with a riding model, the structure did not refine with suitable parameters, and therefore the hydrogen atoms were omitted in the final refinement. Except for atom O2 that was refined with an isotropic displacement parameter, all other atoms were refined with anisotropic displacement parameters.

## Supplementary Material

Crystal structure: contains datablock(s) I. DOI: 10.1107/S2056989018000567/gw2157sup1.cif

Structure factors: contains datablock(s) I. DOI: 10.1107/S2056989018000567/gw2157Isup2.hkl

Supporting information file. DOI: 10.1107/S2056989018000567/gw2157sup3.pdf

CCDC reference: 1571149

Additional supporting information:  crystallographic information; 3D view; checkCIF report

## Figures and Tables

**Table 1:** Experimental details

Crystal data	
Chemical formula	KEu(SO_4_)_2_·H_2_O
*M*_r_	399.18
Crystal system, space group	Trigonal, *P*3_1_21
Temperature (K)	293
*a*, *c* (Å)	6.9065 (2), 12.7802 (5)
*V* (Å^3^)	527.94 (3)
*Z*	3
Radiation type	Mo *K*α
μ (mm^−1^)	10.12
Crystal size (mm)	0.14 × 0.12 × 0.08

Data collection	
Diffractometer	Bruker SMART CCD area detector
Absorption correction	Multi-scan (*SADABS*; Sheldrick, 1996 ▸)
*T*_min_, *T*_max_	0.332, 0.498
No. of measured, independent and observed [*I* > 2σ(*I*)] reflections	3856, 848, 823
*R*_int_	0.032
(sin θ/λ)_max_ (Å^−1^)	0.674

Refinement	
*R*[*F* ^2^ > 2σ(*F* ^2^)], *wR*(*F* ^2^), *S*	0.024, 0.058, 1.07
No. of reflections	848
No. of parameters	56
Δρ_max_, Δρ_min_ (e Å^−3^)	1.16, −0.86
Absolute structure	Flack (1983 ▸)
Absolute structure parameter	−0.02 (3)

Computer programs: *SMART* and *SAINT* (Bruker, 2000 ▸), *SIR92* (Altomare *et al.*, 1993 ▸), *SHELXL97* (Sheldrick, 2008 ▸), *ORTEP-3 for Windows* (Farrugia, 2012 ▸), *CAMERON* (Watkin *et al.*, 1993 ▸) and *PLATON* (Spek, 2009 ▸).

## supplementary crystallographic information

### Crystal data

**Table:**

KEu(SO_4_)_2_·H_2_O	*D*_x_ = 3.767 Mg m^−^^3^
*M**_r_* = 399.18	Mo *K*α radiation, λ = 0.71073 Å
Trigonal, *P*3_1_21	Cell parameters from 848 reflections
Hall symbol: P 31 2"	θ = 3.4–28.6°
*a* = 6.9065 (2) Å	µ = 10.12 mm^−^^1^
*c* = 12.7802 (5) Å	*T* = 293 K
*V* = 527.94 (3) Å^3^	Block like, light yellow
*Z* = 3	0.14 × 0.12 × 0.08 mm
*F*(000) = 558	

### Data collection

**Table:**

Bruker SMART CCD area detector diffractometer	848 independent reflections
Radiation source: fine-focus sealed tube	823 reflections with *I* > 2σ(*I*)
Graphite monochromator	*R*_int_ = 0.032
φ and ω scans	θ_max_ = 28.6°, θ_min_ = 3.4°
Absorption correction: multi-scan (SADABS; Sheldrick, 1996)	*h* = −9→9
*T*_min_ = 0.332, *T*_max_ = 0.498	*k* = −8→9
3856 measured reflections	*l* = −16→17

### Refinement

**Table:**

Refinement on *F*^2^	Secondary atom site location: difference Fourier map
Least-squares matrix: full	H-atom parameters not defined
*R*[*F*^2^ > 2σ(*F*^2^)] = 0.024	*w* = 1/[σ^2^(*F*_o_^2^) + (0.026*P*)^2^ + 3.0791*P*] where *P* = (*F*_o_^2^ + 2*F*_c_^2^)/3
*wR*(*F*^2^) = 0.058	(Δ/σ)_max_ < 0.001
*S* = 1.07	Δρ_max_ = 1.16 e Å^−^^3^
848 reflections	Δρ_min_ = −0.86 e Å^−^^3^
56 parameters	Absolute structure: Flack (1983)
0 restraints	Absolute structure parameter: −0.02 (3)
Primary atom site location: structure-invariant direct methods	

### Special details

**Table:**

Geometry. All s.u.'s (except the s.u. in the dihedral angle between two l.s. planes) are estimated using the full covariance matrix. The cell s.u.'s are taken into account individually in the estimation of s.u.'s in distances, angles and torsion angles; correlations between s.u.'s in cell parameters are only used when they are defined by crystal symmetry. An approximate (isotropic) treatment of cell s.u.'s is used for estimating s.u.'s involving l.s. planes.
Refinement. Refinement of F^2^ against ALL reflections. The weighted R-factor wR and goodness of fit S are based on F^2^, conventional R-factors R are based on F, with F set to zero for negative F^2^. The threshold expression of F^2^ > 2σ(F^2^) is used only for calculating R-factors(gt) etc. and is not relevant to the choice of reflections for refinement. R-factors based on F^2^ are statistically about twice as large as those based on F, and R- factors based on ALL data will be even larger.

### Fractional atomic coordinates and isotropic or equivalent isotropic displacement parameters (Å^2^)

**Table:**

	*x*	*y*	*z*	*U*_iso_*/*U*_eq_	
Eu1	0.56354 (7)	0.56354 (7)	0.5000	0.00972 (12)	
S1	0.5565 (2)	0.5452 (2)	0.25595 (8)	0.0068 (2)	
K1	0.5398 (3)	0.5398 (3)	0.0000	0.0196 (4)	
O4	0.3795 (8)	0.5049 (8)	0.1823 (3)	0.0156 (11)	
O3	0.6104 (7)	0.7411 (7)	0.3231 (3)	0.0142 (9)	
O5	0.4906 (8)	0.3578 (8)	0.3288 (3)	0.0155 (10)	
O1	0.9181 (15)	0.9181 (15)	0.5000	0.068 (4)	
O2	0.7533 (7)	0.5868 (7)	0.1949 (3)	0.0165 (10)*	

### Atomic displacement parameters (Å^2^)

**Table:**

	*U*^11^	*U*^22^	*U*^33^	*U*^12^	*U*^13^	*U*^23^
Eu1	0.01111 (18)	0.01111 (18)	0.00863 (18)	0.0068 (2)	−0.00007 (9)	0.00007 (9)
S1	0.0073 (6)	0.0090 (6)	0.0053 (5)	0.0049 (6)	0.0001 (5)	−0.0008 (4)
K1	0.0254 (8)	0.0254 (8)	0.0167 (8)	0.0191 (10)	0.0004 (4)	−0.0004 (4)
O4	0.013 (2)	0.025 (3)	0.0128 (18)	0.012 (2)	−0.0075 (17)	−0.0030 (18)
O3	0.020 (2)	0.010 (2)	0.0136 (18)	0.008 (2)	−0.0018 (16)	−0.0061 (15)
O5	0.022 (3)	0.016 (2)	0.014 (2)	0.013 (2)	0.0000 (17)	0.0023 (16)
O1	0.024 (4)	0.024 (4)	0.154 (11)	0.012 (4)	−0.017 (3)	0.017 (3)

### Geometric parameters (Å, º)

**Table:**

Eu1—O2^i^	2.425 (4)	S1—K1^i^	3.6182 (15)
Eu1—O2^ii^	2.425 (5)	S1—K1^iv^	3.6579 (19)
Eu1—O4^iii^	2.428 (5)	K1—O5^vii^	2.374 (5)
Eu1—O4^iv^	2.428 (5)	K1—O5^viii^	2.374 (5)
Eu1—O1	2.449 (10)	K1—O3^ix^	2.479 (4)
Eu1—O3^v^	2.514 (4)	K1—O3^x^	2.479 (4)
Eu1—O3	2.514 (4)	K1—O4^xi^	2.538 (4)
Eu1—O5	2.518 (4)	K1—O4	2.538 (4)
Eu1—O5^v^	2.518 (4)	K1—O2	2.830 (4)
Eu1—S1^v^	3.1210 (11)	K1—O2^xi^	2.830 (4)
Eu1—S1	3.1210 (11)	K1—S1^xi^	3.2726 (11)
Eu1—K1^vi^	4.0356 (12)	K1—S1^vii^	3.6182 (15)
S1—O4	1.456 (4)	K1—S1^viii^	3.6182 (15)
S1—O2	1.465 (4)	O4—Eu1^x^	2.428 (5)
S1—O5	1.470 (5)	O3—K1^iv^	2.479 (4)
S1—O3	1.484 (4)	O5—K1^i^	2.374 (5)
S1—K1	3.2726 (11)	O2—Eu1^vii^	2.425 (4)
			
O2^i^—Eu1—O2^ii^	77.6 (2)	K1—S1—K1^i^	105.93 (4)
O2^i^—Eu1—O4^iii^	73.28 (14)	O4—S1—K1^iv^	81.0 (2)
O2^ii^—Eu1—O4^iii^	145.65 (16)	O2—S1—K1^iv^	121.63 (18)
O2^i^—Eu1—O4^iv^	145.65 (17)	O5—S1—K1^iv^	118.38 (17)
O2^ii^—Eu1—O4^iv^	73.28 (14)	O3—S1—K1^iv^	29.66 (17)
O4^iii^—Eu1—O4^iv^	139.6 (2)	Eu1—S1—K1^iv^	72.58 (2)
O2^i^—Eu1—O1	141.21 (10)	K1—S1—K1^iv^	105.03 (4)
O2^ii^—Eu1—O1	141.21 (10)	K1^i^—S1—K1^iv^	143.32 (5)
O4^iii^—Eu1—O1	69.82 (12)	O5^vii^—K1—O5^viii^	128.8 (3)
O4^iv^—Eu1—O1	69.82 (12)	O5^vii^—K1—O3^ix^	76.98 (14)
O2^i^—Eu1—O3^v^	85.25 (14)	O5^viii^—K1—O3^ix^	153.95 (18)
O2^ii^—Eu1—O3^v^	124.29 (13)	O5^vii^—K1—O3^x^	153.95 (18)
O4^iii^—Eu1—O3^v^	71.15 (14)	O5^viii^—K1—O3^x^	76.98 (14)
O4^iv^—Eu1—O3^v^	96.34 (14)	O3^ix^—K1—O3^x^	77.63 (19)
O1—Eu1—O3^v^	72.05 (9)	O5^vii^—K1—O4^xi^	79.99 (15)
O2^i^—Eu1—O3	124.29 (13)	O5^viii^—K1—O4^xi^	113.79 (14)
O2^ii^—Eu1—O3	85.25 (14)	O3^ix^—K1—O4^xi^	69.93 (14)
O4^iii^—Eu1—O3	96.34 (14)	O3^x^—K1—O4^xi^	85.94 (15)
O4^iv^—Eu1—O3	71.15 (14)	O5^vii^—K1—O4	113.79 (14)
O1—Eu1—O3	72.05 (9)	O5^viii^—K1—O4	79.99 (15)
O3^v^—Eu1—O3	144.10 (18)	O3^ix^—K1—O4	85.94 (15)
O2^i^—Eu1—O5	68.88 (14)	O3^x^—K1—O4	69.93 (14)
O2^ii^—Eu1—O5	76.38 (15)	O4^xi^—K1—O4	149.2 (2)
O4^iii^—Eu1—O5	76.45 (15)	O5^vii^—K1—O2	64.32 (13)
O4^iv^—Eu1—O5	119.87 (14)	O5^viii^—K1—O2	99.13 (14)
O1—Eu1—O5	112.47 (11)	O3^ix^—K1—O2	88.93 (13)
O3^v^—Eu1—O5	143.20 (14)	O3^x^—K1—O2	120.92 (13)
O3—Eu1—O5	55.58 (14)	O4^xi^—K1—O2	142.03 (14)
O2^i^—Eu1—O5^v^	76.38 (15)	O4—K1—O2	51.72 (13)
O2^ii^—Eu1—O5^v^	68.88 (14)	O5^vii^—K1—O2^xi^	99.13 (14)
O4^iii^—Eu1—O5^v^	119.87 (14)	O5^viii^—K1—O2^xi^	64.32 (13)
O4^iv^—Eu1—O5^v^	76.45 (15)	O3^ix^—K1—O2^xi^	120.92 (13)
O1—Eu1—O5^v^	112.47 (11)	O3^x^—K1—O2^xi^	88.93 (13)
O3^v^—Eu1—O5^v^	55.58 (14)	O4^xi^—K1—O2^xi^	51.72 (13)
O3—Eu1—O5^v^	143.20 (14)	O4—K1—O2^xi^	142.03 (14)
O5—Eu1—O5^v^	135.1 (2)	O2—K1—O2^xi^	142.94 (19)
O2^i^—Eu1—S1^v^	80.93 (10)	O5^vii^—K1—S1	89.74 (9)
O2^ii^—Eu1—S1^v^	96.53 (10)	O5^viii^—K1—S1	89.12 (10)
O4^iii^—Eu1—S1^v^	96.44 (10)	O3^ix^—K1—S1	87.27 (10)
O4^iv^—Eu1—S1^v^	84.68 (10)	O3^x^—K1—S1	94.81 (10)
O1—Eu1—S1^v^	91.61 (3)	O4^xi^—K1—S1	156.52 (12)
O3^v^—Eu1—S1^v^	27.98 (9)	O4—K1—S1	25.19 (10)
O3—Eu1—S1^v^	154.22 (9)	O2—K1—S1	26.53 (9)
O5—Eu1—S1^v^	149.79 (11)	O2^xi^—K1—S1	151.64 (10)
O5^v^—Eu1—S1^v^	27.67 (11)	O5^vii^—K1—S1^xi^	89.12 (10)
O2^i^—Eu1—S1	96.53 (10)	O5^viii^—K1—S1^xi^	89.74 (9)
O2^ii^—Eu1—S1	80.93 (10)	O3^ix^—K1—S1^xi^	94.81 (10)
O4^iii^—Eu1—S1	84.68 (10)	O3^x^—K1—S1^xi^	87.27 (10)
O4^iv^—Eu1—S1	96.44 (10)	O4^xi^—K1—S1^xi^	25.19 (10)
O1—Eu1—S1	91.61 (3)	O4—K1—S1^xi^	156.52 (12)
O3^v^—Eu1—S1	154.22 (9)	O2—K1—S1^xi^	151.64 (10)
O3—Eu1—S1	27.98 (9)	O2^xi^—K1—S1^xi^	26.53 (9)
O5—Eu1—S1	27.67 (11)	S1—K1—S1^xi^	177.34 (9)
O5^v^—Eu1—S1	149.79 (11)	O5^vii^—K1—S1^vii^	15.34 (9)
S1^v^—Eu1—S1	176.78 (5)	O5^viii^—K1—S1^vii^	132.36 (15)
O2^i^—Eu1—K1^vi^	69.92 (10)	O3^ix^—K1—S1^vii^	73.36 (10)
O2^ii^—Eu1—K1^vi^	141.97 (10)	O3^x^—K1—S1^vii^	144.15 (11)
O4^iii^—Eu1—K1^vi^	36.57 (10)	O4^xi^—K1—S1^vii^	64.67 (11)
O4^iv^—Eu1—K1^vi^	127.33 (11)	O4—K1—S1^vii^	127.34 (11)
O1—Eu1—K1^vi^	73.44 (2)	O2—K1—S1^vii^	79.43 (9)
O3^v^—Eu1—K1^vi^	35.80 (9)	O2^xi^—K1—S1^vii^	88.26 (10)
O3—Eu1—K1^vi^	129.45 (10)	S1—K1—S1^vii^	104.28 (3)
O5—Eu1—K1^vi^	108.36 (11)	S1^xi^—K1—S1^vii^	74.79 (3)
O5^v^—Eu1—K1^vi^	84.42 (10)	O5^vii^—K1—S1^viii^	132.36 (15)
S1^v^—Eu1—K1^vi^	59.86 (3)	O5^viii^—K1—S1^viii^	15.34 (9)
S1—Eu1—K1^vi^	121.20 (3)	O3^ix^—K1—S1^viii^	144.15 (11)
O4—S1—O2	107.5 (2)	O3^x^—K1—S1^viii^	73.36 (10)
O4—S1—O5	112.4 (3)	O4^xi^—K1—S1^viii^	127.34 (11)
O2—S1—O5	111.0 (3)	O4—K1—S1^viii^	64.67 (11)
O4—S1—O3	110.6 (3)	O2—K1—S1^viii^	88.26 (10)
O2—S1—O3	110.1 (3)	O2^xi^—K1—S1^viii^	79.43 (9)
O5—S1—O3	105.2 (2)	S1—K1—S1^viii^	74.79 (3)
O4—S1—Eu1	130.45 (19)	S1^xi^—K1—S1^viii^	104.28 (3)
O2—S1—Eu1	121.99 (18)	S1^vii^—K1—S1^viii^	140.69 (8)
O5—S1—Eu1	52.70 (17)	S1—O4—Eu1^x^	144.3 (3)
O3—S1—Eu1	52.64 (16)	S1—O4—K1	106.9 (2)
O4—S1—K1	47.92 (18)	Eu1^x^—O4—K1	108.69 (16)
O2—S1—K1	59.63 (17)	S1—O3—K1^iv^	133.1 (2)
O5—S1—K1	129.05 (18)	S1—O3—Eu1	99.4 (2)
O3—S1—K1	125.43 (18)	K1^iv^—O3—Eu1	107.82 (15)
Eu1—S1—K1	177.55 (5)	S1—O5—K1^i^	139.4 (2)
O4—S1—K1^i^	106.2 (2)	S1—O5—Eu1	99.6 (2)
O2—S1—K1^i^	91.13 (18)	K1^i^—O5—Eu1	117.07 (16)
O5—S1—K1^i^	25.29 (17)	S1—O2—Eu1^vii^	140.9 (3)
O3—S1—K1^i^	128.50 (17)	S1—O2—K1	93.8 (2)
Eu1—S1—K1^i^	76.13 (3)	Eu1^vii^—O2—K1	104.89 (15)

Symmetry codes: (i) −*y*+1, *x*−*y*, *z*+1/3; (ii) *x*−*y*, −*y*+1, −*z*+2/3; (iii) *x*−*y*+1, −*y*+1, −*z*+2/3; (iv) −*y*+1, *x*−*y*+1, *z*+1/3; (v) *y*, *x*, −*z*+1; (vi) −*x*+*y*+1, −*x*+1, *z*+2/3; (vii) −*x*+*y*+1, −*x*+1, *z*−1/3; (viii) −*x*+1, −*x*+*y*+1, −*z*+1/3; (ix) −*x*+1, −*x*+*y*, −*z*+1/3; (x) −*x*+*y*, −*x*+1, *z*−1/3; (xi) *y*, *x*, −*z*.
