# Supplementary material for: Synthesis and crystal structure of a new polymorph of potassium europium(III) bis(sulfate) mono­hydrate, KEu(SO4)2·H2O
Source: Acta Crystallogr E Crystallogr Commun. 2018 Jan 26;74(Pt 2):242–5. doi: 10.1107/S2056989018000567 (PMC5956346; doi:10.1107/S2056989018000567)
Supplement: Supplementary file 3 [file e-74-00242-sup3.pdf]

Supporting Information

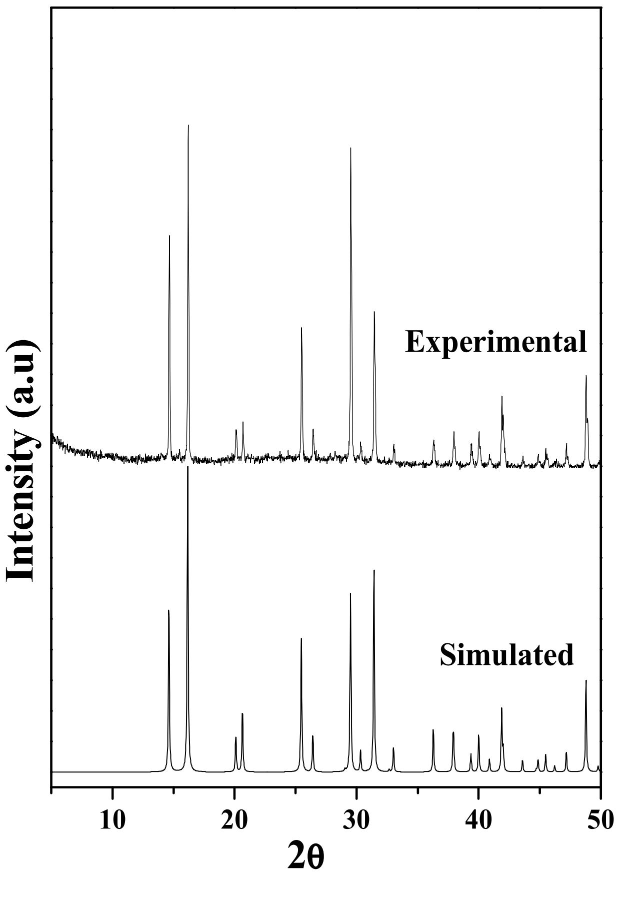

PXRD pattern of compound **1**

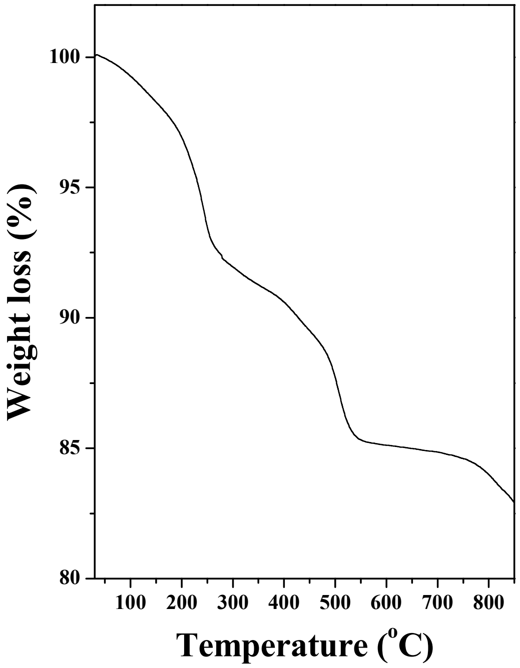

Weight loss (%)
100
95
90
85
80
100
200
300
400
500
600
700
800
Temperature (°C)

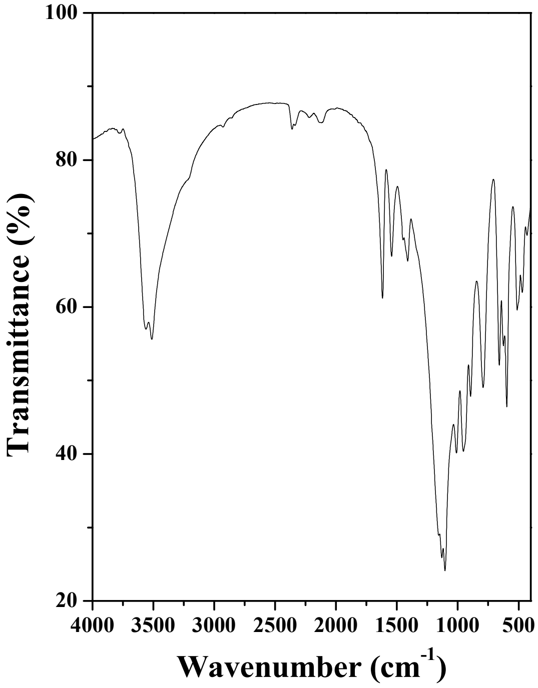

100
80
60
40
20
Transmittance (%)
4000
3500
3000
2500
2000
1500
1000
500
Wavenumber (cm-1)

IR and TGA Study

The IR study shows that the compound contains the water molecule and sulfate units. The sharp peak in the region of 3550-3500 cm^{-1} corresponds to O-H stretching frequency for water molecule. The sharp peak at 1100 cm^{-1} corresponds to S-O bond vibrational frequency. The region below 700 cm^{-1} corresponds to metal-oxygen vibrational frequency. The TGA plot indicated that the compound **1** shows a steady weight loss of 16 %. The weight loss corresponds to dissociation of water and sulfate moiety. One can predict the final product is mixture of metal oxide after thermal analysis.

Atomic coordinates and isotropic displacement parameters (in Å [2])

Atom	Wyck.	x	y	z	U
Eu1	3*b*	0.56354(7)	0.56354(7)	0.50000	0.00972(12)
K1	*3a*	0.5398(3)	0.5398(3)	0.0000	0.0196(4)
S1	6*c*	0.5565(2)	0.5452(2)	0.25595(8)	0.0068(2)
O1	3*b*	0.9181(15)	0.9181(15)	0.5000	0.068(4)
O2	6*c*	0.7533(7)	0.5868(7)	0.1949(3)	0.0165(10)
O3	6*c*	0.6104(7)	0.7411(7)	0.3231(3)	0.0142(9)
O4	6*c*	0.3795(8)	0.5049(8)	0.1823(3)	0.0156(11)
O5	6*c*	0.4906(8)	0.3578(8)	0.3288(3)	0.0155(10)

Anisotropic displacement parameters (in Å [2])

Atoms	U_{11}	U_{22}	U_{33}	U_{23}	U_{13}	U_{12}
Eu1	0.01111(18)	0.01111(18)	0.00863(18)	0.00007(9)	-0.00007(9)	0.0068(2)
K1	0.0254(8)	0.0254(8)	0.0167(8)	-0.0004(4)	0.0004(4)	0.0191(10)
S1	0.0073(6)	0.0090(6)	0.0053(5)	-0.0008(4)	0.0001(5)	0.0049(6)
O1	0.024(4)	0.024(4)	0.154(11)	0.017(3)	-0.017(3)	0.012(4)
O3	0.020(2)	0.010(2)	0.0136(18)	-0.0061(15)	-0.0018(16)	0.008(2)
O4	0.013(2)	0.025(3)	0.0128(18)	-0.0030(18)	-0.0075(17)	0.012(2)
O5	0.022(3)	0.016(2)	0.014(2)	0.0023(16)	0.0000(17)	0.013(2)

Selected geometric parameters (Å, °)

Bond length	(Å)	Bond length	(Å)
Eu(1)-O(1)	2.449(10)	K(1)-O(3)	2.479(4)
Eu (1)-O(2)	2.425(4)	K(1)-O(4)	2.538(5)
Eu (1)-O(3)	2.514(4)	S(1)-O(2)	1.465(4)
Eu (1)-O(4)	2.428(5)	S(1)-O(3)	1.484(4)
Eu (1)-O(5)	2.518(4)	S(1)-O(4)	1.456(4)
K(1)-O(5)	2.374(5)	S(1)-O(5)	1.470(5)
K(1)-O(2)	2.830(4)		
**Bond angle**	**(deg)**	**Bond angle**	**(deg)**
O(2)-Eu(1)-O(2)#1	77.6(2)	O(5)-K(1)-O(5)#1	128.8(3)
O(2)- Eu (1)-O(4)	145.65(16)	O(5)-K(1)-O(3)	153.95(18)
O(2)-Eu(1)-O(4)#1	73.28(14)	O(5)-K(1)-O(3)#1	76.98(14)
O(4)-Eu(1)-O(4)#1	139.6(2)	O(3)-K(1)-O(3)#1	77.63(19)
O(1)-Eu(1)-O(2)	141.21(10)	O(4)-K(1)-O(5)	113.79(14)
O(1)- Eu (1)-O(4)	69.82(12)	O(4)-K(1)-O(5)#1	79.99(15)
O(2)- Eu (1)-O(3)	124.29(13)	O(3)-K(1)-O(4)	69.93(14)
O(3)- Eu (1)-O(2)#1	85.25(14)	O(3)-K(1)-O(4)#1	85.94(15)
O(3)- Eu (1)-O(4)	71.15(15)	O(4)-K(1)-O(5)	79.99(15)
O(3)- Eu (1)-O(4)#1	96.34(14)	O(4)-K(1)-O(3)	149.2(2)
O(1)- Eu (1)-O(3)	72.05(9)	O(5)-K(1)-O(2)#1	64.32(13)
O(3)- Eu (1)-O(5)	143.20(14)	O(2)-K(1)-O(5)#1	99.13(14)
O(5)- Eu (1)-O(2)	68.88(14)	O(2)-K(1)-O(3)	120.92(13)
O(5)- Eu (1)-O(4)	76.45(15)	O(2)-K(1)-O(4)#1	51.72(13)
O(5)- Eu (1)-O(4)#1	119.87(14)	O(3)-S(1)-O(5)	105.2(2)
O(1)- Eu (1)-O(4)	112.45(11)	O(3)-S(1)-O(4)	110.6(3)
O(2)- Eu (1)-O(4)#1	143.29(15)	O(5)-S(1)-O(4)	112.4(3)
O(3)- Eu (1)-O(4)	55.45(14)	O(3)-S(1)-O(2)	110.1(3)
O(5)- Eu (1)-O(4)	68.90(15)	O(5)-S(1)-O(2)	111.0(3)
O(5)- Eu (1)-O(4)#1	76.40(16)	O(4)-S(1)-O(2)	107.5(2)
